# Cinematic Amputations in Italian Hospitals

**Published:** 1918-09

**Authors:** 


### ORTHOPEDICS.

**Cinematic Amputations in Italian Hospitals.** A Report to the Chief Surgeon, A. E. F., by Major William S. BAER, M. R. C., and Captain Philip. D. WILSON, M. R. C.

*Historical* : The idea of cinematization was first conceived by Dr. Vanghetti in 1897-8, as a result of his observations in the Italian Abyssinian war. In this conflict the Abyssinians cut off the left hand at the wrist joint of those Italians under their dominion who proved traitorous to them. In consequence of this, all the muscle of the fore-arm remained intact. This fact led him to the study of the utilization of the stump, as an active force in producing movements of the artificial limb. Experiments were carried out by him on chickens, on account of their prehensile power. From these experiments, he was able to demonstrate that the muscles of the stump could be utilized for active mobility.

The first operation on a human subject was performed by Professor Ceci of Pisa about 1900. This work was soon followed by that of Sauerbruck of Germany, and by De Francesco and Galleazzi of Italy. While reports from Germany and Austria indicate that similar work is being done there, Italy alone of the Allies is showing much activity along these lines.

*General Principles of Cinematic Amputations.*

The general principles of Cinematization are based both on anatomical and physiological considerations.

Under our present methods of amputation the muscle power of the stump is lost. Under the cinematic method the muscle power is utilized.

All motion in a normal limb is accomplished by the antagonism of muscle groups. This physiological principle is utilized in cinematic amputations. It is accomplished by connecting at the end of stump the antagonistic muscles, or by giving them artificial insertion into the prosthetic apparatus.

*Types of Cinematization.* There are various types of cinematization. The surgical procedure varies according to the method by which the muscular attachment is made, thereby producing function in the artificial limb. The attachments are called motors. The motors most commonly employed are :

1. The club.
2. The loop.
3. The tendon tunnel.
4. The muscle tunnel.
5. Pseudo-arthrosis of the terminal stump segment.

1. The club is a projecting mass of muscle or tendon covered with skin, which being lassoed can be attached to the artificial limb.

2. The loop is formed by the junction of antagonistic tendons or muscles, completely surrounded by skin. Owing to its ringlike character it offers a convenient means of attachment to the artificial limb.

3. The tendon tunnel is a tunnel of skin surrounded by a loop of tendon and lying beneath the skin.

4. The muscle tunnel is a tunnel of skin passing through the belly of a muscle.

5. The pseudo-arthrosis motor is a small segment of terminal bone, with its muscles and tendons intact, which has been separated at its proximal end and rendered mobile. In this way it conveys its power to the artificial limb.

*Centers of Cinematization in Italy.* The following institutions constitute the chief centers of this work:

*Institute Rizzoli, Bologna: — Major Putti, Captain Delitala.*

All types of motor are selected to meet the needs of the individual case. Particular pains are taken to secure muscle antagonism by suture of muscles also to obtain free gliding surfaces. Examination of various cases after cinematization demonstrated that by this method a definite force could be obtained which could be utilized for actuating artificial limbs.

In connection with this hospital there is an excellent prosthetic workshop so that progress both from the surgical and mechanical side can proceed simultaneously. This seems very important.

Professor Putti, contrary to the opinion of most men, believes that the value of a stump is not altogether dependent upon its length, that it is the degree of impairment of the nerves controlling the muscles that is the most important. Before proceeding to a cinematic operation he applies three tests to determine whether the case is applicable.

1. Clinical method — Trying the development of the muscles, their mobility, and their contractibility by palpation.

2. Physiological method — Analyzing the power by movement of the stumps against resistance.

3. Anatomical method — Considering the amount of active muscle remaining and the skin available.

Only when when these tests are satisfactory does he proceed to operate.

II. *Ospedale Clinicale, Florence: — General Burci.*

Burci believes that the simplest possible motor is the best and that 25 o/o of all amputés are suitable for the application of this method.

Conditions necessary to have good result are:

1. Good muscles with good tonicity, not much damaged.
2. Non adherent tendons.
3. Sufficient skin to cover ring or club.

III. *Doctor Julian Vanghetti, Empoli, Florence.*
*Italian Red Cross.*

Doctor Vanghetti, although not a surgeon, is the acknowledged author of cinematization. He believes at the present stage that simple motors are best, but with the greater perfection of prosthesis a larger number of motors will be used. He says that it has been absolutely demonstrated that motors increase in strength with years rather than diminish.

IV. *General L. Bononio, General Medical Inspector.*

Bononio believes that the method is applicable and most useful in the upper extremity and that the single motor is best. He also thinks that amputations at the front should be performed by the flapless, plane section method. This causes the least possible traumatism to the muscle and thus tends to preserve its gliding action.

V. *Ospedale Ancili, Mantua.*

The director laid great stress upon the necessity of a flapless amputation at the front followed immediately by the application of traction to the skin in order to prepare the case for cinematization.

VI. *Ospedale Centrale Principale, Verona: — Captain G. Pieri.*

Pieri has performed 34 operations of this nature and has written several articles on the subject. He stands as one of the chief exponents of the method.

He states that all amputations at the front should be done from a physiological rather than an anatomical point of view. This means the preservation of muscle innervation. The saving of skin and the integrity of skin flaps by making incisions in such a way as to conserve their circulation. He calls this hypocinematization. He states that bone may be sacrificed if thereby muscular function is increased. The use of multiple motors is advocated and he prefers either the club, or an unusually thick loop. According to his opinion motors may be applied to all parts of the limbs save the upper third of the thigh and leg.

VII. *Ospedale Croce Rossa, Chiari: — Major Pellegrini.*

As a preliminary to cinematization, Doctor Pellegrini insists upon the early application of efficacious traction to the skin and for this has invented several ingenious types of apparatus to be used by ambulatory cases which at the same time permit of motion of the joint above the stump. By this method he is able to secure healing in most cases of flapless amputations. He insists that secondary amputations are practically never necessary. By preparing his motors above the end of the stump he is able to secure good surgical results at an early date.

VIII. *Pio Instituto dei Rachitichi, Milan: — Lt.-Col. Galeazzi.*

Colonel Galeazzi believes it is more difficult to obtain a good surgical result than is generally claimed. He states that tendon motors give less good results than muscle motors on account of their tendency to adhere. Single motors are the best. The question of obtaining sufficient skin is very important.

He lays stress upon the necessity of developing prosthetic apparatus before practical results can be obtained, especially in the upper extremity. At present the most that can be secured in the arm is the opening and closing of the prosthetic apparatus. However, on account of the lamentable state of arm amputés, at present, anything that offers possibilities such as this should be developed.

### Conclusions.

The following conclusions are based upon personal examination of 35 cases of cinematization after operation, and reports of 165 cases representing the total number of operations performed by the surgeons visited, together with a thorough discussion of the subject with many surgeons interested in it.

1. At present cinematization is in an experimental stage. The results so far obtained have been of little practical value in improving the working efficiency of the amputé. Psychologically and esthetically it has been a distinct step in the betterment of the individual.

2. The results obtained are, however, of great importance in opening up a new field for future development. Based, as it is, upon sound physiological principles it seems reasonable to suppose that progress along these lines will bring results of great practical value.

3. At present much more progress has been made along surgical lines than along mechanical lines in the development of cinematization. The muscular power obtained by the surgical procedures has not been well utilized on account of the lack of mechanical efficiency in the apparatus.

4. In order to obtain further progress prosthesis and surgery, as applied to cinematization must go hand in hand. If prosthesis cannot adapt itself to the surgical procedure, then the latter must adapt itself to the prothesis. For this reason they must both be under the same direction.

5. Cinematization is at present more applicable to the upper than to the lower extremity. On account of the more delicate and more complex functions of the upper limb as compared with those of the lower limb, present types of apparatus are much less efficient for the former than for the latter. The voluntary control which cinematization offers makes its use especially applicable to the upper.

6. The present development of cinematization, particularly in reference to prosthesis makes it important to use the single type of motor. With the further development of prothesis it is logical to suppose that a greater number of motors will be demanded. Whatever the kind of motor used, it should not interfere with the proper adjustment of the prothetic apparatus.

7. In order that American amputés may be able to benefit by future progress along cinematic lines, it is necessary that amputations at the front should be performed in a definite manner. The flapless amputation should be the method of choice. Muscles, skin and bone should be conserved and primary suture should only be done for amputés when these structures are not sacrificed.

8. Application of traction to the skin should always be made as a part of the treatment. The importance of this procedure cannot be over-estimated.

*Recommendations.* 1. Primitive suture of amputations should never be performed, when it involves the sacrifice of bone, muscles, or skin.

2. As a rule the guillotine or flapless method of amputation is advised, thus preserving the gliding effect of the muscles, length of bone, and skin.

3. Traction should be applied *immediately* to the skin after amputation, to preserve and increase its length and to retain the tenacity of the muscles.

4. All cases of amputation should be evacuated as soon as possible to an amputation center. That is, as soon as they are able to travel after their operation or amputation has been performed.

5. Cinematic operation should only be performed by surgeons who have direct control of a prosthetic workshop. Unless the workshop is associated with a hospital for the amputes, the patient will not derive the results which are possible.

6. An amputation center with a prosthetic workshop should be established immediately in the A. E. F. to care for those amputés

7. One or more amputation centers should be established immediately in the United States for those cases which it is wise to return immediately.

